# Crohn’s disease: failure of a proprietary fluorescent in situ hybridization assay to detect *M. avium* subspecies *paratuberculosis* in archived frozen intestine from patients with Crohn’s disease.

**DOI:** 10.1186/s13104-020-04947-0

**Published:** 2020-02-24

**Authors:** Robert J. Greenstein, Liya Su, Peter S. Fam, Brooke Gurland, Paul Endres, Sheldon T. Brown

**Affiliations:** 1 Department of Surgery, James J. Peters Veterans Affairs Medical Center Bronx, New York, USA; 2 Laboratory of Molecular Surgical Research, James J. Peters Veterans Affairs Medical Center Bronx, New York, USA; 3 Mental Illness Research Education and Clinical Center (MIRECC), James J. Peters, Veterans Affairs Medical Center Bronx, New York, USA; 4 Colorectal Surgery, Stanford University School of Medicine, Stanford CA, USA; 5 Department of Pathology, Icahn School of Medicine at Mount Sinai New York, New York, USA; 6 Infectious Disease Section, James J. Peters Veterans Affairs Medical Center Bronx, New York, USA; 7 Department of Medicine, Icahn School of Medicine at Mt. Sinai. New York, New York, USA

**Keywords:** In situ hybridization, *Mycobacterium avium* subspecies *paratuberculosis*, Mycobacteria, Crohn disease, Johne disease

## Abstract

**Objectives:**

Although controversial, there is increasing concern that Crohn’s disease may be a zoonotic infectious disease consequent to a mycobacterial infection. The most plausible candidate is *M. avium* subspecies *paratuberculosis* (MAP) that is unequivocally responsible for Johne’s disease in ruminants. The purpose of this study was to evaluate a proprietary (Affymetrix™ RNA view^®^) fluorescent in situ hybridization (FISH) assay for MAP RNA. Non-identifiable intestine from patients with documented Crohn’s disease was assayed according to the manufacturer’s instructions and with suggested modifications. Probes were custom designed for MAP and human β-actin (as the eukaryotic housekeeping gene) from published genomes.

**Results:**

Repetitively, false positive signal was observed in our “No-Probe” negative control. Attempts were made to correct this according to the manufacturer’s suggestions (by modifying wash solutions, using recommended hydrochloric acid titration and different fluorescent filters). None prevented false positive signal in the “No-Probe” control. It is concluded that when performed according to manufactures instruction and with multiple variations on the manufactures recommended suggestions to correct for false positive signal, that the Affymetrix™ RNA view^®^ cannot be used to detect MAP in pre-frozen resected intestine of humans with Crohn’s disease.

## Introduction

Johne’s disease [1] a chronic wasting intestinal infection in animals is caused by *M. avium* subspecies *paratuberculosis* (MAP). Viable MAP is found in the human food chain including pasteurized milk in the US [2] and Europe and chlorinated municipal water [3]. There is increasing concern that MAP may be zoonotic. [4] and might be an etiological trigger for Crohn’s disease, an affliction evocative of Johne’s disease [3, 5–7] The gold standard of diagnosis of Johne’s disease is culture of MAP [8]. In animals with Johne’s disease, this is a reliable, but time-consuming process. Multiple other diagnostic modalities exist for detecting mycobacteria in general [8–11] and MAP in particular. [12–16] Following the detection of putative MAP, confirmation usually requires the identification of the DNA sequence IS900 which is unique to MAP [17].

In humans with Crohn’s disease, Ziehl [18]-Neelsen [19] staining, usually visualized at × 400 magnification, does not identify mycobacteria [6, 7]. Using oil-immersion microscopy (×1000 magnification) *M. avium* was identified in Crohn’s disease. [20] Although MAP has been cultured from humans with Crohn’s disease [21], this is difficult, few laboratories can do so [22–26], and up to 18 months may be required for the organism to reconstitute its cell wall [21]. The detection of MAP DNA does not signify that the organism was viable [27]. In contrast, detecting MAP RNA implies viability [3]. It would therefore be of use to develop an assay that reliably and rapidly identifies MAP RNA in possibly infected intestine.

We herein report on our attempts to develop a fluorescent in situ hybridization (FISH) assay of MAP RNA, using two proprietary RNA amplification techniques. One is specifically designed for tissue (Affymetrix™ RNA view^®^. View ISH Tissue Assay Kit 96 2-Plex. Thermo Fisher Catalog Number: QVT0013.) The second is a product specifically designed for isolated cell assays (ViewRNA ISH Cell Assay Kit; Invitrogen by Thermo Fisher Scientific: Catalog Number: QVC0001.) In this report we studied multiple tissues. Initially, intestine from patients with Crohn’s disease that had been snap frozen in liquid nitrogen and stored at − 80 ℃ until processed for assay. Human intestine studied included frozen sections, routinely imbedded paraffin tissue and autopsy specimens. As further controls, intestine from ruminants with Johne’s disease were evaluated. Finally, we evaluated human buffy coat circulating white blood cells (WBC’s.)

## Main text

This study was approved by the Research & Development Committee at the VAMC Bronx NY (0720-06-038.) The methods and results with bovine ileal intestinal tissue, with and without Johne’s disease have been published [28]. The non-identifiable tissue from individuals with and without Crohn’s disease were archived samples that had been stored at − 80 ℃.

The tissue and assay were handled in an identical manner to that of the published bovine study [28], with one exception. Previously, at our request Affymetrix had generated probes that were species specific from published gene sequences. In this study the housekeeping gene was Human Specific β-actin (Affymetrix Catalog # VA6-10506-1 Probe type 6) As in the previous study for MAP, an Affymetrix generated probe designed using the published sequence [17]. (Affymetrix name: *M. tuberculosis* Is900: Cat # VF1 19496: Lot # 195634523: Probe type 1.) Previously, the house keeping gene for ruminants was bovine β-actin (Bos Taurus actb: NCBI Reference Sequence: NM_173979.3 (Affymetrix name: Bos Taurus Actb: Cat# VF6 20062: Lot # 200642784: Probe Type 6.) We also use 16S Bacterial, Probe type 6, Cat # VF-6-16576-01: 16 S *E. coli*, Probe type 1 Cat # VF1-19200-01: 16S *Mycobacterium tuberculosis* Probe type 1, Cat # VF1-16224-01: Human β-Actin (ACTB Human) Probe type 1, Cat # VA1-10351-01: Bovine β-Actin (ACTB BOB TAURUS)Probe type 1 Cat# VF1-20959-01: IS 900 (*M. avium* subspecies paratuberculosis) Probe type 6, Cat # VF6-20958-01: IS6110 (*Mycobacterium tuberculosis*) Probe type 1, Cat # VF1-6000090-01: and Human GAPD (glycaraldehyde-3-phosphate dehydrogenase) (As an additional house-keeping gene) Probe type 6 Cat # VA6-100337-01. All these probes are proprietary to Affymetrix.

**Fig. 1:**
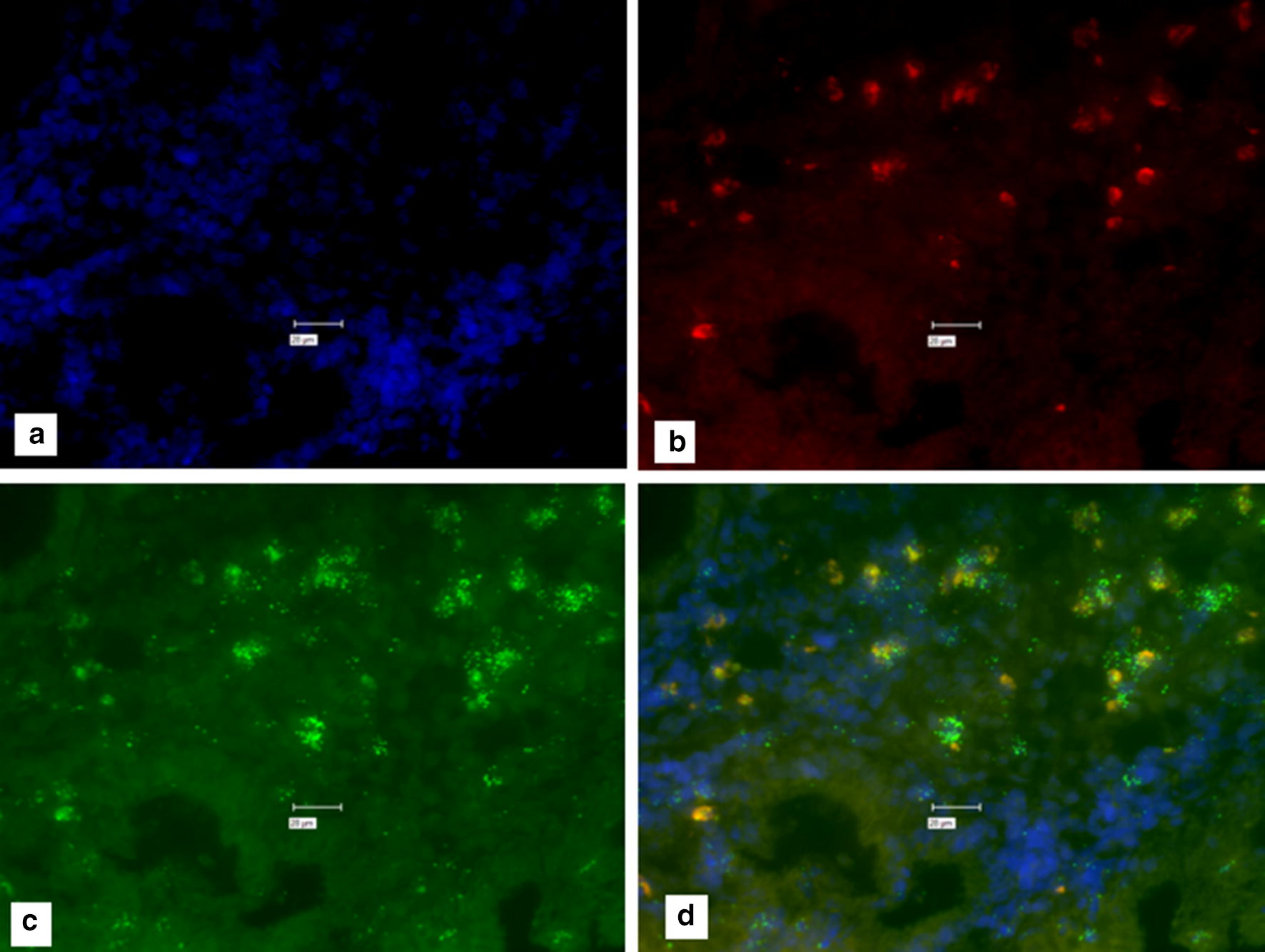
From a sample of intestine from a patient with Crohn’s disease. **a** DAPI; **b** Texas Red (IS900 an insertion sequence unique to *M. avium* subspecies *paratuberculosis*.); **c** Cy-5 (Human β-actin; a housekeeping control gene) **d** composite of **a**–**c**. Note “positive” signal in the “with-probe” in Fig. 1 panels **b**–**d**. Marker bars in µm indicates magnification of × 40

**Fig. 2:**
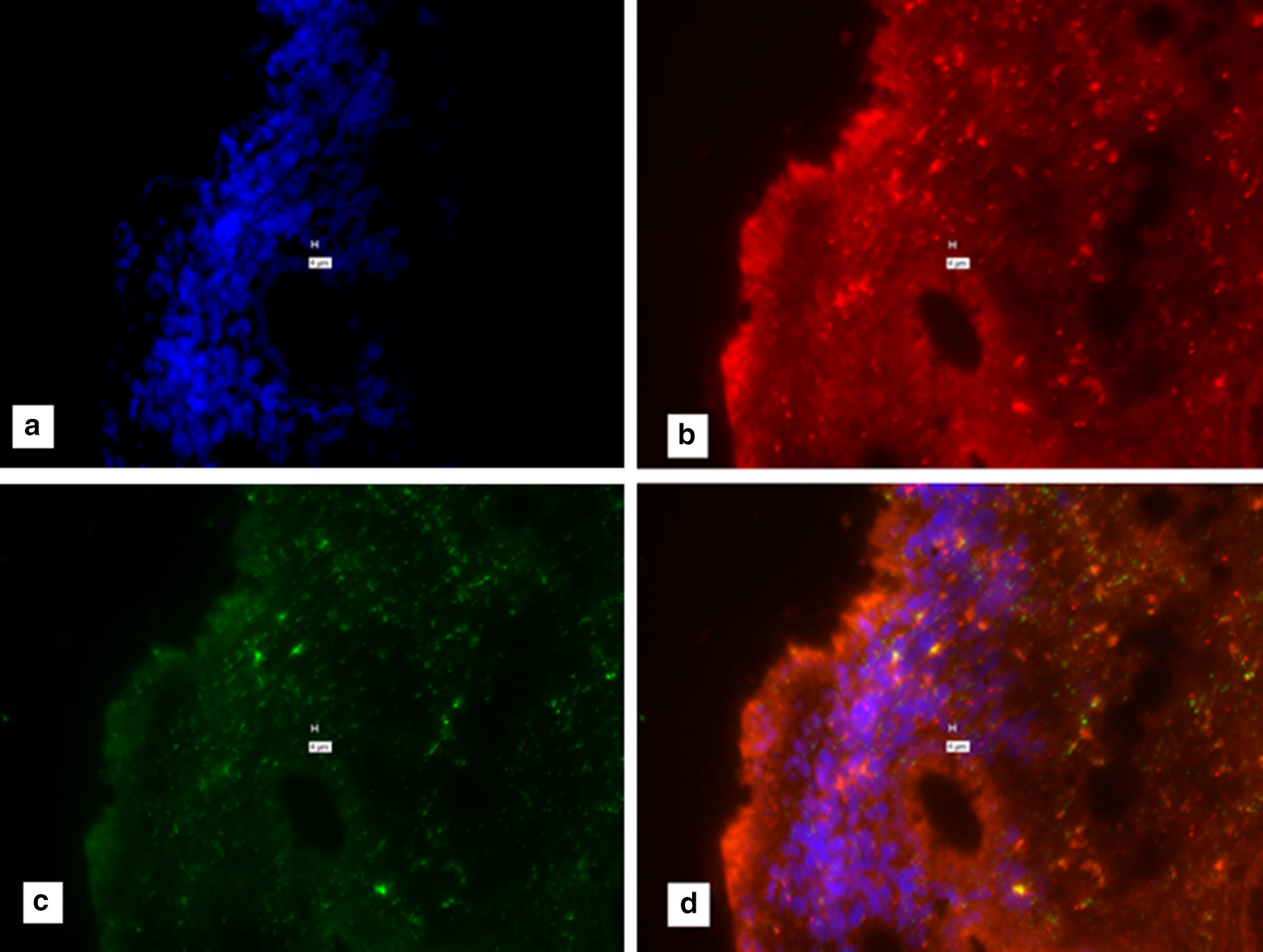
No-Probe Control for Fig. 1. A composite of four images of the same section, of human Crohn’s disease intestine. Processed identically as in Fig. 1, during the same experiment, but NO-probes were added during the hybridization step. **a** DAPI; **b** Texas Red; **c** Cy-5 (**d** composite of **a**–**c**.) Note “positive” signal in Fig. 1**b**–**d**. Marker bars in µm indicates magnification of × 40

**Fig. 3:**
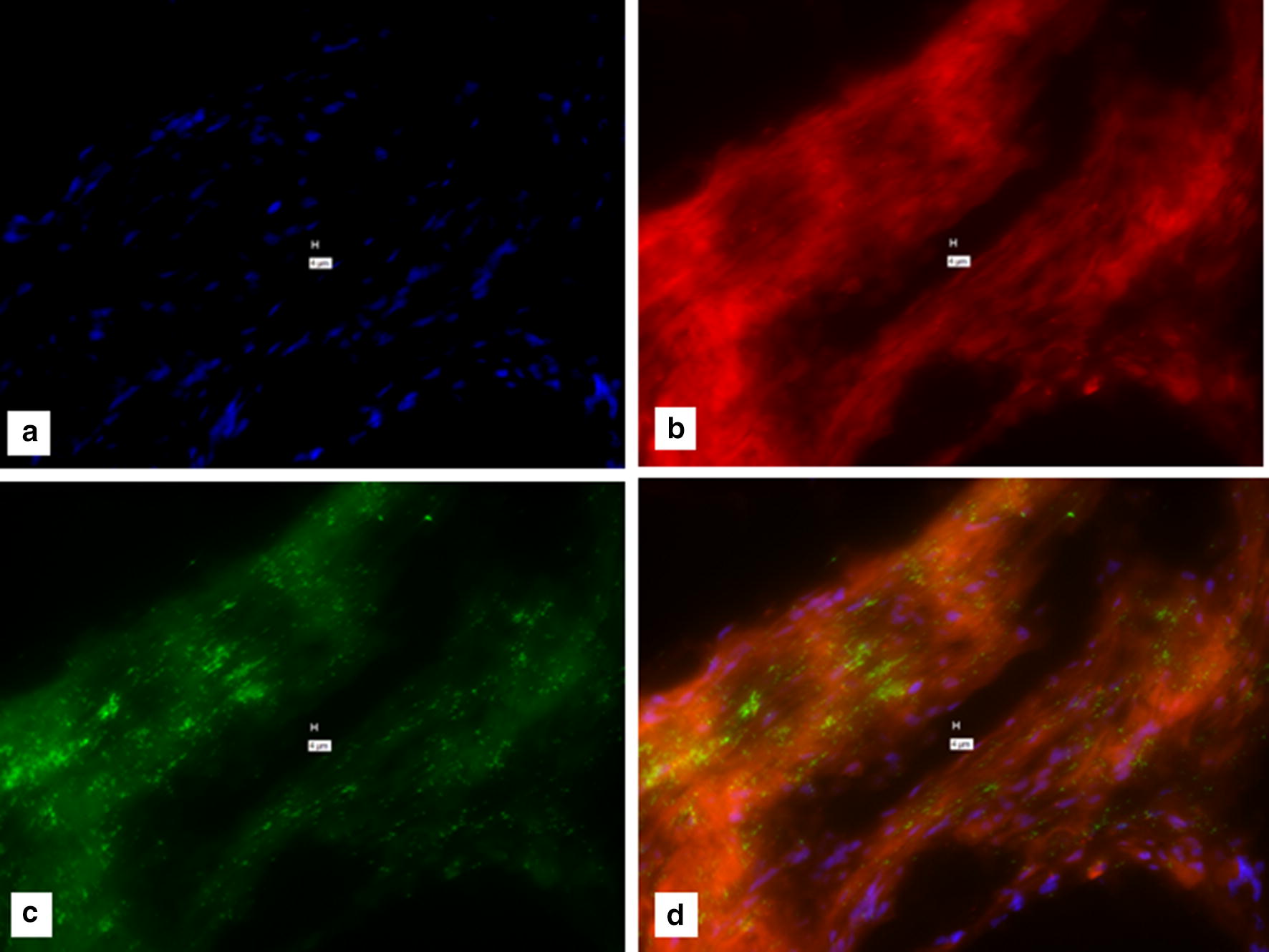
“No-probe” negative for a 15-min exposure to 0.2 M HCl. **a** DAPI; **b** Texas Red; **c** Cy-5 (**d** composite of **a**–**c**.) Note the “positive” signal in this “No-probe” control for Fig. 3**b**–**d**. Marker bars in µm indicates magnification of × 40. (See Additional file 1 for the With-Probe control for this Figure.) The incubation with HCl is recommended to correct for false positive due to endogenous alkaline phosphatase in the tissue being studied

All human tissues were non-identifiable. Different from our previous study [28], on human samples the house keeping genes were human specific β-actin probe and human GAPD (glycaraldehyde-3-phosphate dehydrogenase). Initial human intestinal studies were carried out identically as published [28]. Repetitively a clear background could not be observed in the control slide, from which probes had been excluded during the Probe Set Hybridization steps (Fig. 1). The Affymetrix ViewRNA™ ISH Tissue 2-Plex Assay instructions, suggests pretreating tissue with HCl to prevent false positive signal (Affymetrix: Protocol Guide for RNA in situ Hybridization. Troubleshooting for high background. Page 71.) As previously, multiple attempts were made to obtain a clear background. Figure 1 shows “positive signal With-Probe with an HCL wash. However, Fig. 2 likewise shows “positive” signal when No-Probes are used. Figure 3 shows “positive” signal when No-Probes are used with a 15 min 0.2 M HCl wash.

Multiple additional experiments were performed in an attempt to obtain truly negative No-Probe controls. We have previously presented some of our data concerning both time and concentration titration of exposure to HCl. Additional file 1, (the With Probe Control for Fig. 3) shows the results of treating with 0.2 M HCl for 15 min. Next, we attempted to see whether we could study human intestinal autopsy samples. Because we were using archived frozen samples, we attempted to study other human intestinal. Additional file 2 shows a comparison of routine surgical resected intestine (Panel A) with autopsy colon (Panel B, obtained approximately 24 h following death.) The profound autolysis precludes autopsy tissue being used. We then studied archived paraffin imbedded samples of human intestine. Additional file 3 shows de-paraffinized human tissue probed with 16S E. Coli and human β-actin. The positive signal noted is also seen in the No-Probe control (Additional file 4. See Panel “C”.) We concluded that paraffinized tissue cannot be used in this assay. We next examine tissue sent for frozen section. Additional file 5 was probed with 16S bacterial probe and human β-actin. The No-Probe control (Additional file 6) has abundant false positive signal (see white square marker Panel “C”. We conclude that fresh tissue taken from frozen sections also exhibit false positivity. Frozen bovine tissue was then probed (Additional file 7) with Bovine β-actin and IS900. The No-Probe control for exhibits abundant false positive signal (Additional file 8 Panels “B”, “C” & “D”.) We conclude that the false positive signal previously shown [28] is reproducible.

False positive signal may be due to endogenous alkaline phosphatase activity. This can be identified by staining with Fast Blue Substrate (Additional file 9). Affymetrix Technical support states that this may be corrected by incubating in 0.2MHCl for 15 min [28]. We present previously unpublished data of our titrations at 25 min with probes (Additional file 10) and without probes (Additional file 11), and increased HCL concentration (0.4 M) with probes (Additional file 12). The No-Probe Control is Additional file 13. We conclude that HCl treatment did not correct false positive signal.

Because circulating white blood cells (WBC’s) may harbor mycobacteria, we next studied WBC’s obtained from buffy coat obtained using a Ficoll gradient. Additional file 14 is with and Additional file 15 is without probes. Although the false positive signal is less pronounced in isolated WBC’s it is still present. We attempted to use RNA Paxgene^®^, but full hemolysis occurred, so no WBC’s could be obtained. As an alternative, we evaluated DNA Paxgene^®^ to draw blood. Additional file 16 is with probes and Additional file 17 is its No-Probe control. Both DNA Paxgene ^®^ tubes give poor quality images. As the No-Probe controls showing false positive signal, we conclude that DNA Paxgene ^®^ has no role in establishing a WBC FISH assay.

We next studied two house-keeping genes human β-actin and human GAPD (glycaraldehyde-3-phosphate dehydrogenase.) For this we used a different kit specifically designed for singles cells (ViewRNA ISH Cell Assay Kit ^®^.) Clean positive signal with both probes is seen in Additional file 18. They are always colocalized to DAPI positive regions; indicating that the signal is associated with white blood cells. In contrast, False positive signal is only seen in the No-Probe control (Additional file 19). Specifically, the Cy-5 field shows no background whatsoever. Uniquely in these studies, but only with the single Cell Assay Kit, we consider that this ViewRNA ISH Cell Assay Kit ^®^, on WBC’s may represent genuine signal.

We then evaluated the utility of RNALater^®^ on WBC’s. Blood was stored at 4 ℃ for 24 h prior to isolating the buffy coat. Additional file 20 present the with probe and Additional file 21 its No-Probe control. False positive signal is present the No-Probe control.

Because of the encouraging data from Additional files 18 and 19, we then evaluated the Single cell Affymetrix assay on bovine Johne intestine both with (Additional file 22) and without (Additional file 23) probes. Because of abundant positive signal in the No-Probe control (Additional file 23 panels “B”, “C” and “D”) we conclude that the Single cell Affymetrix system ViewRNA ISH Cell Assay Kit ^®^ CANNOT be used to study intestinal tissue in lieu of Affymetrix™ RNA view^®^ Tissue assay kit ^®^.

During our efforts to obviate false positive signal we repetitively contacted the Technical staff at ThermoFisher Affymetrix. They provided us with a Rat Kidney Control Kit that contained three slides. Two had reciprocal probe sets (types 1 and 6.) The third slide was ThermoFisher’s “No-Probe” control. In Additional file 24 we show the Affymetrix provided No-Probe control with false positive signal (See panels’ B”, “C” and “D.”)

We have previously shown that this false positive “No-probe” signal cannot be ascribed to contamination of the negative control slide by probes during the post hybridization wash or obtained by increasing the stringency or duration of the HCL pretreatment, nor did using different filters (TritC for Texas-Red, and for Cy-5 a custom recommended filter set “Hope” [28] recommended by the technical staff at Affymetrix prevent false positive signal.

A proprietary FISH assay has been performed according to the recommended conditions of the vendor. Purportedly positive signal was detected for both MAP (IS900) and our eukaryotic housekeeping gene, human β-actin. However, repetitively the “No-Probe” negative control for a given experiment showed obviously false “positive” signal.

It is concluded that when the assay is performed according to the Affymetrix ViewRNA™ ISH Tissue 2-Plex Assay recommended instructions, it cannot be used for FISH studies to identify the RNA of MAP on previously frozen Crohn’s disease intestine.

There were compelling reasons to initiate these studies. MAP is known to cause Johne disease. It MAY be causative in both Crohn disease and multiple sclerosis. These concerns are addressed in the introduction to our three manuscripts. We stand by our conclusions that QVT0013 cannot be used to determine the presence or absence of MAP in the three diseases studied under the conditions under which they were studied. We concluded that by publishing our findings others in the scientific community would waste neither the time, energy nor expense in performing identical studies

## Limitations

In this study we asked a binary question. Is MAP present or absent in a given sample of intestine obtained from humans with Crohn’s disease? Especially when the target is expected to be in low abundance, any background may result in a false positive interpretation and is unacceptable. In contrast, when a change in gene expression is being quantified for example comparing normal with inflamed tissue, a low FISH signal to noise background may be acceptable. Accordingly, our conclusions apply only to frozen intestinal tissue where we are attempting to identify presumably low abundance MAP and not to other scientific investigations.

The ViewRNA™ ISH Tissue 2-Plex Assay is designed to study tissue, not isolated cells. Encouragingly, when circulating WBC’s are studied using the ViewRNA ISH Cell Assay Kit^®^, possibly reliable data have been obtained. However, this ViewRNA ISH Cell Assay Kit cannot be used on tissue; only WBC’s. Although the ThermoFisher provided No-Probe slides had positive signal, it was not stated whether these had been frozen prior to processing. Our studies were not performed on fresh tissue. Therefore, our conclusions should only be applied to frozen, not fresh, intestine.

## Supplementary information

**Additional file 1.** A “With -Probe” Control for Fig. 3. Crohn tissue treated with 15-min exposure to 0.2 M HCl, prior to hybridizing with probes. A = DAPI; B = Texas Red (IS900); C = Cy-5 (Human β-actin) D = composite of A, B and C. Marker bars in µm indicates magnification of × 100.

**Additional file 2.** Comparison of surgical specimen that had been immediately placed in formaldehyde (A), with post -mortem colon (B). Note the complete cellular disintegration in the post-mortem specimen (B). We conclude that autopsy tissue cannot be evaluated for this assay. Stain is Hematoxylin & Eosin × 10.

**Additional file 3.** With probes: Human gut from paraffin block. A = DAPI; B = Texas Red (Probe is 16S E Coli Type 1); C = Cy-5 (Probe is Human β-actin; Type 6) D = composite of A, B and C. Note “positive” signal in panel “C” (White arrow.) Marker bars in µm indicates magnification of × 40.

**Additional file 4.** No-Probe control for Additional file 3: Figure S3: Human gut from paraffin block. A = DAPI; B = Texas Red (No-Probe); C = Cy-5 (No-Probe) D = composite of A, B and C. Note “apparent positive “signal in panel “C” (White arrows.) Indicating false positive signal. Marker bars in µm indicates magnification of × 40.

**Additional file 5.** With probes: Human intestine from frozen tissue. A = DAPI; B = Texas Red (Probe is Human β-actin; Type 1 Red); C = Cy-5 (Probe is 16S E Bacteria Type 6 Green) D = composite of A, B and C. Note apparent positive signal in panel “B” (White arrow) and generalized in “C.) Marker bars in µm indicates magnification of × 40.

**Additional file 6.** No-Probe control for Additional file 5: Figure S5. Human intestine from frozen tissue. A = DAPI; B = Texas Red (No-Probe); C = Cy-5 (No-Probe) D = composite of A, B and C. Note apparent positive signal in white square and generalized in panel “C”. Indicating false positive signal. Marker bars in µm indicates magnification of × 40.

**Additional file 7.** With probes: Frozen Bovine intestine with Johne disease. A = DAPI; B = Texas Red (Probe is Bovine β-actin; Type 6 Green); C = Cy-5 (Probe is IS 900 Type 1 Red) D = composite of A, B and C. Note scattered apparent “positive “signal in panel “B” & arrows. Marker bars in µm indicates magnification of × 40.

**Additional file 8.** No-Probe control for Additional file 7: Figure S7. Frozen Bovine intestine with Johne disease. A = DAPI; B = Texas Red. C = Cy-5 D = composite of A, B and C. Note scattered apparent “positive” signal in panels “B” “C” and “D” Indicating false positive signal. Marker bars in µm indicates magnification of × 40.

**Additional file 9.** Fast Blue Substrate. This is to determine the presence of Alkaline Phosphatase, that is associated with false positive signal. No Probe are used. Fluorescence indicates presence of abundant Alkaline Phosphatase. Frozen Johne tissue. Marker bars in µm indicates magnification of × 40.

**Additional file 10.** With probes: HCl treatment to mitigate endogenous alkaline phosphatase. Previously unpublished images of 0.2 M HCl exposed for 25 min. Frozen Bovine intestine with Johne disease. A = DAPI; B = Texas Red (Probe is IS 900 Type 1 Red); C = Cy-5 Probe is Bovine β-actin; Type 6 Green) (D = composite of A, B and C. Note scattered apparent “positive “signal in panel “B” and arrows. Marker bars in µm indicates magnification of × 40.

**Additional file 11.** No-Probe control for Additional file 10 Figure S10 Frozen Bovine intestine with Johne disease. A = DAPI; B = Texas Red. C = Cy-5 D = composite of A, B and C. Note scattered apparent “positive” signal in panels “B” “C” and “D”. Indicating that longer exposure to 0.2 M HCL thatn the recommended 15 min do not resolve the endogenous alkaline problem. Marker bars in µm indicates magnification of × 40.

**Additional file 12.** With probes: HCl treatment to mitigate endogenous alkaline phosphatase. Previously unpublished images of 0.4 M HCl exposed for 15 min. Frozen Bovine intestine with Johne disease. A = DAPI; B = Texas Red (Probe is IS 900 Type 1 Red); C = Cy-5 Probe is Bovine β-actin; Type 6 Green) (D = composite of A, B and C. Note scattered apparent “positive “signal in panels “B”, “C” and “D”. Marker bars in µm indicates magnification of × 40.

**Additional file 13.** No-Probe control for Additional file 12 Figure S12. Frozen Bovine intestine with Johne disease. A = DAPI; B = Texas Red. C = Cy-5 D = composite of A, B & C. Note scattered apparent “positive” signal in panels “B” “C” and “D”. Indicating that exposure to more concentrated 0.4 M HCL than the recommended 0.2 M. HCl do not resolve the endogenous alkaline problem. Marker bars in µm indicates magnification of × 40.

**Additional file 14.** With probes: Human circulating buffy coat white blood cells. A = DAPI; B = Texas Red (Probe is I Human β-actin; Type 1 Red); C = Cy-5 Probe is IS 900 Type 6 Green) (D = composite of A, B and C. Note scattered apparent “positive “signal in panels “B”, “C” and “D”. Marker bars in µm indicates magnification of × 100.

**Additional file 15.** No-Probe control for Additional file 14. Human circulating buffy coat white blood cells. A = DAPI; B = Texas Red (No-Probe) C = Cy-5 No-Probe) (D = composite of A, B and C. Note scattered apparent “positive “signal in panels “B”, and “D”. Although less pronounced than in Additional file 14, false positive signal is detectable in this No-Probe control. Marker bars in µm indicates magnification of × 100.

**Additional file 16.** With probes: Drawn into DNA Paxgene^®^ tubes. Human circulating buffy coat white blood cells. A = DAPI; B = Texas Red (Probe is I Human β-actin; Type 1 Red); C = Cy-5 Probe is IS 900 Type 6 Green) (D = composite of A, B and C. Note scattered apparent “positive “signal in panels “B”, “C” and “D”. Marker bars in µm indicates magnification of × 40.

**Additional file 17.** No-Probes control for Additional file 16: Drawn into DNA Paxgene^®^ tubes. Human circulating buffy coat white blood cells. A = DAPI; B = Texas Red; C = Cy-5 (D = composite of A, B and C. Note scattered apparent “positive “signal in panels “B”, “C” and “D”. Arrow identifies false positive signal in Panel “B”. There is more abundant false positivity in “C”, Cy-5. Marker bars in µm indicates magnification of × 40.

**Additional file 18.** With probes: Human circulating buffy coat white blood cells. Specimens were processed immediately. A = DAPI; B = Texas Red (Probe is Human b-actin; Type 1 Red); C = Cy-5 Probe is Human GAPD (glycaraldehyde-3-phosphate dehydrogenase: Type 6 Green) (D = composite of A, B and C. Note scattered “positive “signal in panels “B”, “C” and “D”. These are always associated with DAPI positive regions, indicating that the signal is associated with white blood cells. They may be genuine and not be spurious background signal. Marker bars in µm indicates magnification of × 40.

**Additional file 19.** No-Probes control for Additional file 18: Specimens were processed immediately. Human circulating buffy coat white blood cells. A = DAPI; B = Texas Red; C = Cy-5 Probe (D = composite of A, B and C. Note scattered apparent “positive “signal in panels “B”, “C” and “D”. There is zero false positive signal with the Cy-5 filter (Panel “C”.) There is some signal associated with Texas Red (Panels B and D.) It is of significance that this, predominantly, is NOT associated with DAPI positive regions. This indicates that reliable negative control with circulating WBC’s may be achievable with the Affymetrix ViewRNA ISH Cell Assay Kit^®^; Invitrogen by Thermo Fisher Scientific, Catalog Number: QVC0001. Magnification is x 40.

**Additional file 20.** With probes: With RNA Later^®^. Specimens were stored at 4 °C for 24 h before being processed. Human circulating buffy coat white blood cells. A = DAPI; B = Texas Red (Probe is Human β-actin; Type 1 Red); C = Cy-5 Probe is Human GAPD (glycaraldehyde-3-phosphate dehydrogenase: Type 6 Green) (D = composite of A, B and C. Note scattered apparent “positive “signal in panels “B”, and “D”. Marker bars in µm indicates magnification of × 100.

**Additional file 21.** No-probe control for Additional file 20: With RNA Later^®^. Specimens were stored at 4 °C for 24 h before being processed. Human circulating buffy coat white blood cells. A = DAPI; B = Texas Red (Probe is Human β-actin; Type 1 Red); C = Cy-5 Probe is Human GAPD (glycaraldehyde-3-phosphate dehydrogenase: Type 6 Green) (D = composite of A, B & C. Note scattered apparent “positive “signal in panels “B”, “C” and “D”. Marker bars in µm indicates magnification of × 100.

**Additional file 22.** Affymetrix Single Cell Kit with Johne intestine. With Probes. A = DAPI; B = Texas Red (Probe is Bovine β-actin; Type 1 Red); C = Cy-5 (Probe is IS 900 Type 6 Green) (D = composite of A, B and C.) Purportedly positive signal is seen in panels “B”, “C” and “D”. Marker bars in µm indicates magnification of × 100.

**Additional file 23.** Affymetrix Single Cell Kit with Johne intestine. No-Probe control for Additional file 22. A = DAPI; B = Texas Red C = Cy-5 (D = composite of A, B and C.) Abundantly false positive signal is seen in panels “B”, “C” and “D”. This indicates that the Affymetrix Single Cell kit is of no utility studying archived frozen intestine. Marker bars in µm indicates magnification of × 100.

**Additional file 24.** Affymetrix in house controls, provided to our laboratory. Slides were fully processed prior to arrival in our laboratory. The only action taken by us, was to read them. Panel A is Bright Field. Panels B = Texas Red C = Cy-5 (D = composite of B and C.) Tissues is rat fetal kidney. False positive signal is seen in panels “B” “C” & “D”.

## Data Availability

Data presented in manuscript.

## Abbreviations

MAP: *M. avium* subspecies *paratuberculosis*
FISH: Fluorescent in situ hybridization
HCL: Hydrochloric acid
