# Supplementary figures and images for: Crohn’s disease: failure of a proprietary fluorescent in situ hybridization assay to detect M. avium subspecies paratuberculosis in archived frozen intestine from patients with Crohn’s disease
Source: BMC Res Notes. 2020 Feb 24;13:96. doi: 10.1186/s13104-020-04947-0 (PMC7038517; doi:10.1186/s13104-020-04947-0)

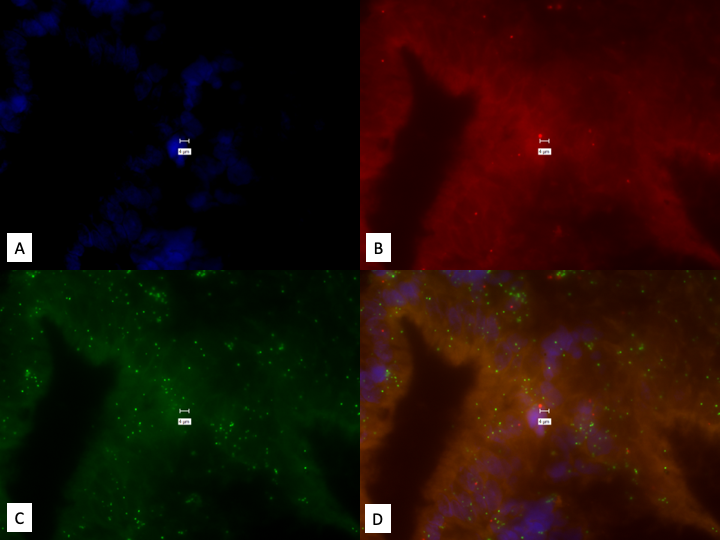

Supplement: Supplementary file 1 — Additional file 1. A “With -Probe” Control for Fig. 3. Crohn tissue treated with 15-min exposure to 0.2 M HCl, prior to hybridizing with probes. A = DAPI; B = Texas Red (IS900); C = Cy-5 (Human β-actin) D = composite of A, B and C. Marker bars in µm indicates magnification of × 100. [file 13104_2020_4947_MOESM1_ESM.tiff]

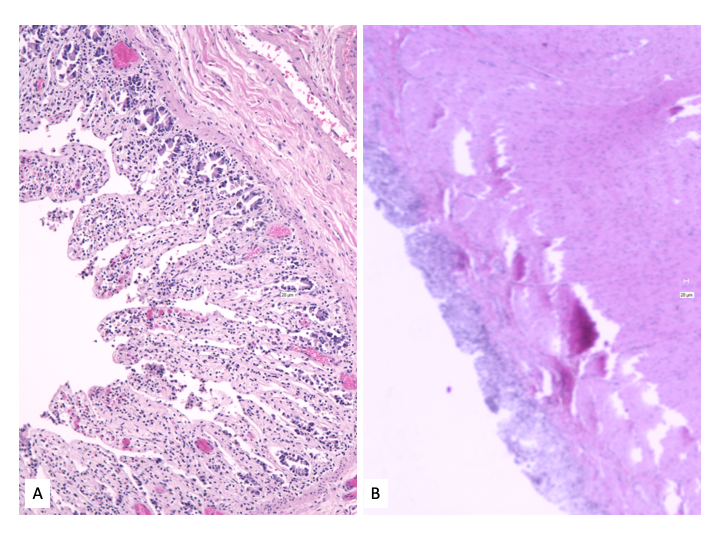

Supplement: Supplementary file 2 — Additional file 2. Comparison of surgical specimen that had been immediately placed in formaldehyde (A), with post -mortem colon (B). Note the complete cellular disintegration in the post-mortem specimen (B). We conclude that autopsy tissue cannot be evaluated for this assay. Stain is Hematoxylin & Eosin × 10. [file 13104_2020_4947_MOESM2_ESM.tiff]

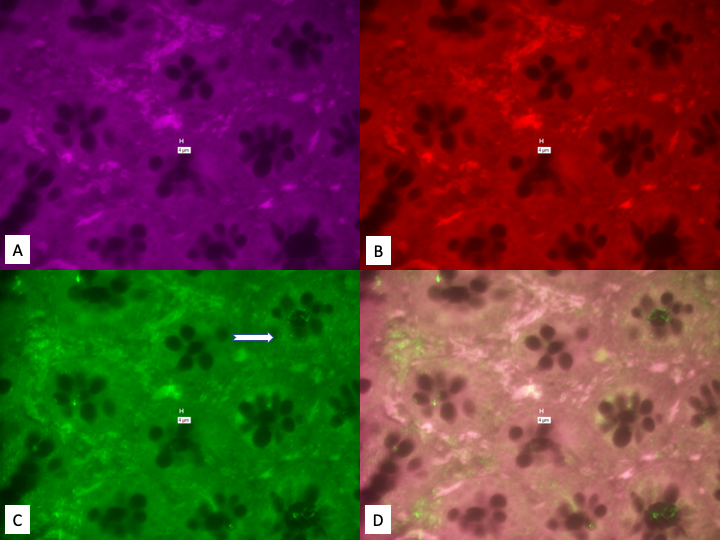

Supplement: Supplementary file 3 — Additional file 3. With probes: Human gut from paraffin block. A = DAPI; B = Texas Red (Probe is 16S E Coli Type 1); C = Cy-5 (Probe is Human β-actin; Type 6) D = composite of A, B and C. Note “positive” signal in panel “C” (White arrow.) Marker bars in µm indicates magnification of × 40. [file 13104_2020_4947_MOESM3_ESM.tiff]

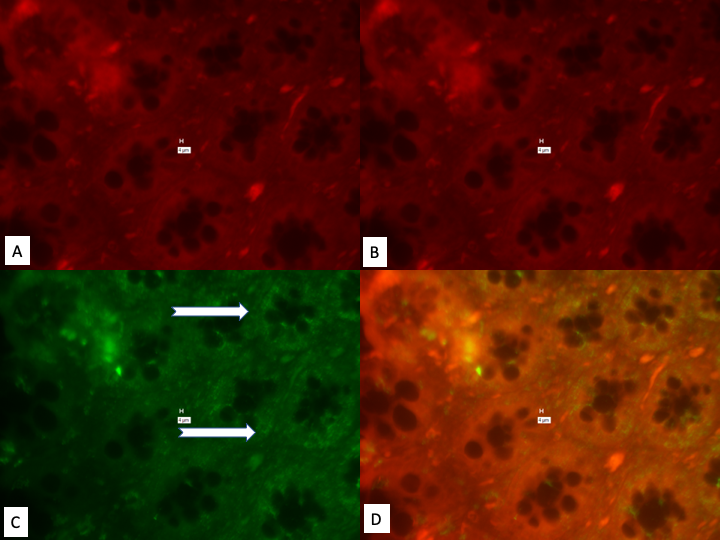

Supplement: Supplementary file 4 — Additional file 4. No-Probe control for Additional file 3: Figure S3: Human gut from paraffin block. A = DAPI; B = Texas Red (No-Probe); C = Cy-5 (No-Probe) D = composite of A, B and C. Note “apparent positive “signal in panel “C” (White arrows.) Indicating false positive signal. Marker bars in µm indicates magnification of × 40. [file 13104_2020_4947_MOESM4_ESM.tiff]

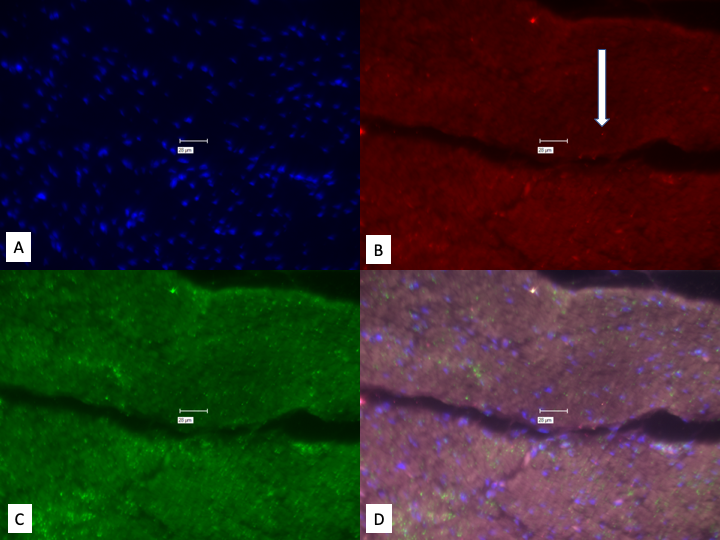

Supplement: Supplementary file 5 — Additional file 5. With probes: Human intestine from frozen tissue. A = DAPI; B = Texas Red (Probe is Human β-actin; Type 1 Red); C = Cy-5 (Probe is 16S E Bacteria Type 6 Green) D = composite of A, B and C. Note apparent positive signal in panel “B” (White arrow) and generalized in “C.) Marker bars in µm indicates magnification of × 40. [file 13104_2020_4947_MOESM5_ESM.tiff]

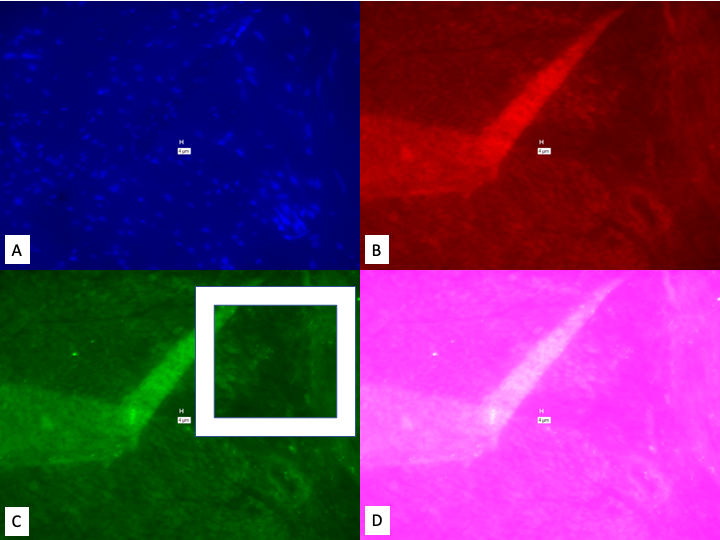

Supplement: Supplementary file 6 — Additional file 6. No-Probe control for Additional file 5: Figure S5. Human intestine from frozen tissue. A = DAPI; B = Texas Red (No-Probe); C = Cy-5 (No-Probe) D = composite of A, B and C. Note apparent positive signal in white square and generalized in panel “C”. Indicating false positive signal. Marker bars in µm indicates magnification of × 40. [file 13104_2020_4947_MOESM6_ESM.tiff]

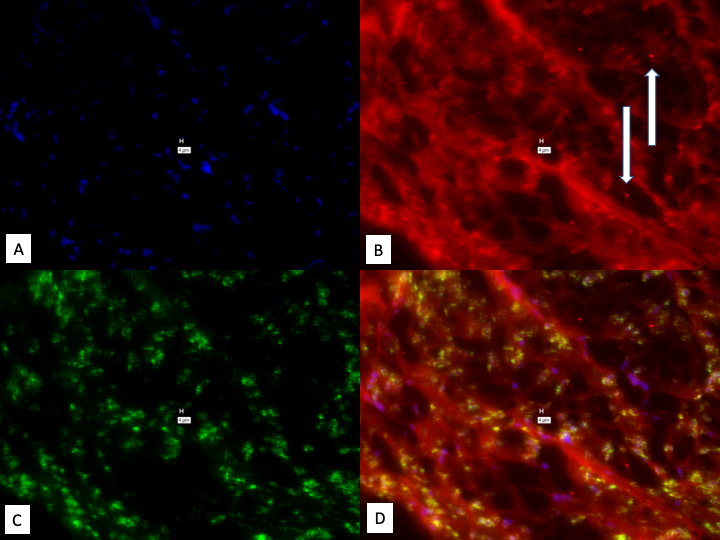

Supplement: Supplementary file 7 — Additional file 7. With probes: Frozen Bovine intestine with Johne disease. A = DAPI; B = Texas Red (Probe is Bovine β-actin; Type 6 Green); C = Cy-5 (Probe is IS 900 Type 1 Red) D = composite of A, B and C. Note scattered apparent “positive “signal in panel “B” & arrows. Marker bars in µm indicates magnification of × 40. [file 13104_2020_4947_MOESM7_ESM.tiff]

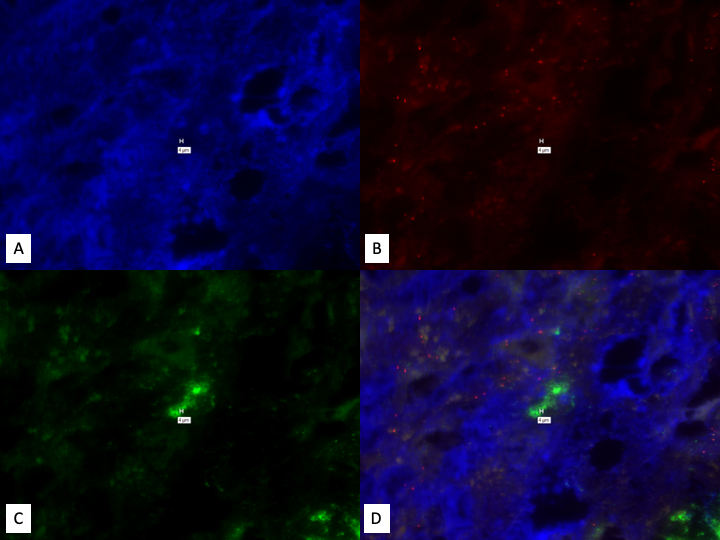

Supplement: Supplementary file 8 — Additional file 8. No-Probe control for Additional file 7: Figure S7. Frozen Bovine intestine with Johne disease. A = DAPI; B = Texas Red. C = Cy-5 D = composite of A, B and C. Note scattered apparent “positive” signal in panels “B” “C” and “D” Indicating false positive signal. Marker bars in µm indicates magnification of × 40. [file 13104_2020_4947_MOESM8_ESM.tiff]

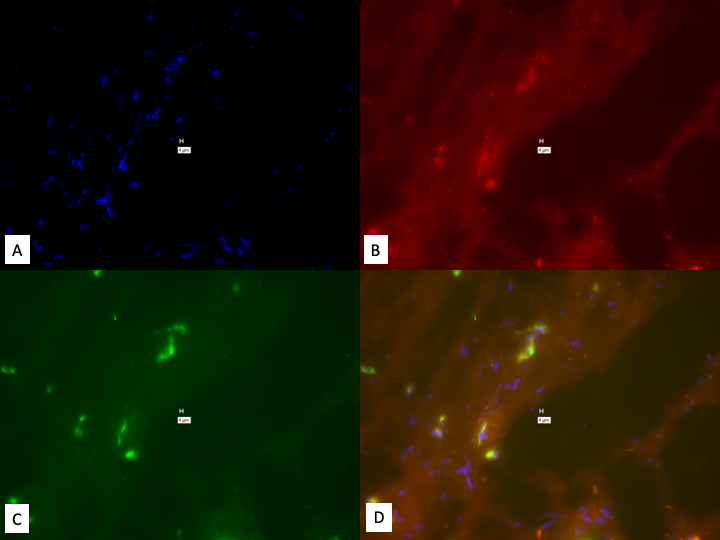

Supplement: Supplementary file 9 — Additional file 9. Fast Blue Substrate. This is to determine the presence of Alkaline Phosphatase, that is associated with false positive signal. No Probe are used. Fluorescence indicates presence of abundant Alkaline Phosphatase. Frozen Johne tissue. Marker bars in µm indicates magnification of × 40. [file 13104_2020_4947_MOESM9_ESM.tiff]

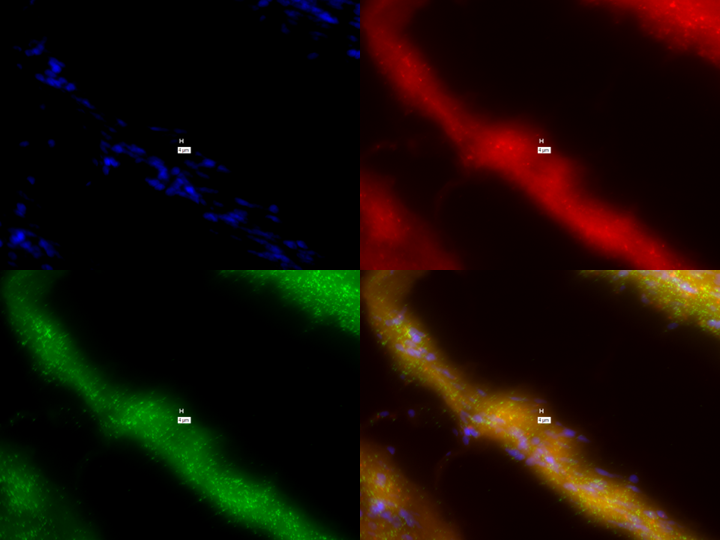

Supplement: Supplementary file 10 — Additional file 10. With probes: HCl treatment to mitigate endogenous alkaline phosphatase. Previously unpublished images of 0.2 M HCl exposed for 25 min. Frozen Bovine intestine with Johne disease. A = DAPI; B = Texas Red (Probe is IS 900 Type 1 Red); C = Cy-5 Probe is Bovine β-actin; Type 6 Green) (D = composite of A, B and C. Note scattered apparent “positive “signal in panel “B” and arrows. Marker bars in µm indicates magnification of × 40. [file 13104_2020_4947_MOESM10_ESM.tiff]

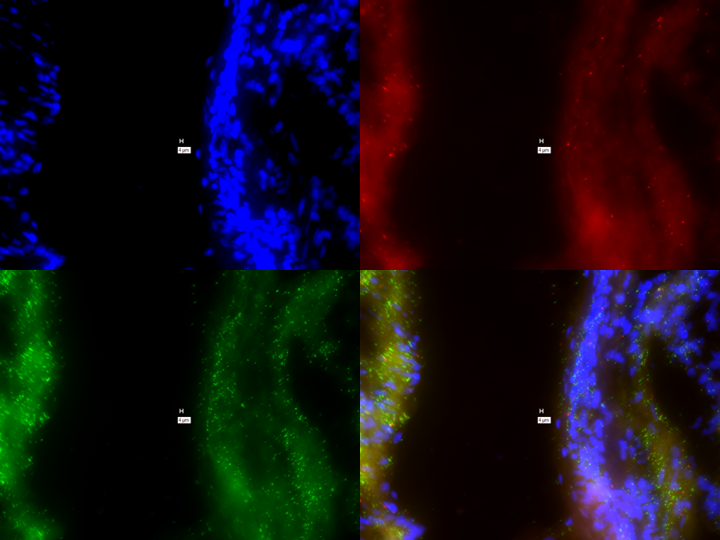

Supplement: Supplementary file 11 — Additional file 11. No-Probe control for Additional file 10 Figure S10 Frozen Bovine intestine with Johne disease. A = DAPI; B = Texas Red. C = Cy-5 D = composite of A, B and C. Note scattered apparent “positive” signal in panels “B” “C” and “D”. Indicating that longer exposure to 0.2 M HCL thatn the recommended 15 min do not resolve the endogenous alkaline problem. Marker bars in µm indicates magnification of × 40. [file 13104_2020_4947_MOESM11_ESM.tiff]

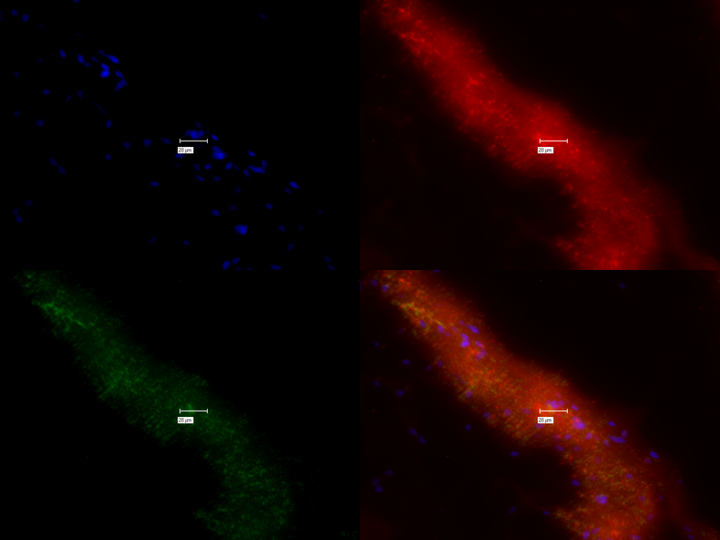

Supplement: Supplementary file 12 — Additional file 12. With probes: HCl treatment to mitigate endogenous alkaline phosphatase. Previously unpublished images of 0.4 M HCl exposed for 15 min. Frozen Bovine intestine with Johne disease. A = DAPI; B = Texas Red (Probe is IS 900 Type 1 Red); C = Cy-5 Probe is Bovine β-actin; Type 6 Green) (D = composite of A, B and C. Note scattered apparent “positive “signal in panels “B”, “C” and “D”. Marker bars in µm indicates magnification of × 40. [file 13104_2020_4947_MOESM12_ESM.tiff]

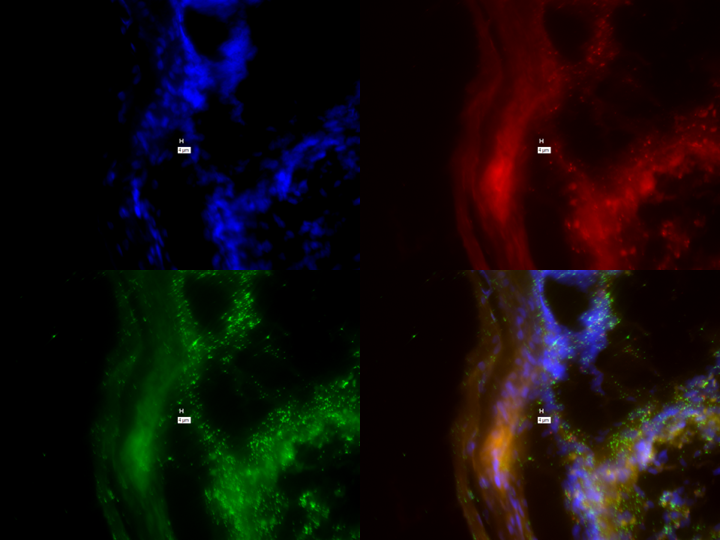

Supplement: Supplementary file 13 — Additional file 13. No-Probe control for Additional file 12 Figure S12. Frozen Bovine intestine with Johne disease. A = DAPI; B = Texas Red. C = Cy-5 D = composite of A, B & C. Note scattered apparent “positive” signal in panels “B” “C” and “D”. Indicating that exposure to more concentrated 0.4 M HCL than the recommended 0.2 M. HCl do not resolve the endogenous alkaline problem. Marker bars in µm indicates magnification of × 40. [file 13104_2020_4947_MOESM13_ESM.tiff]

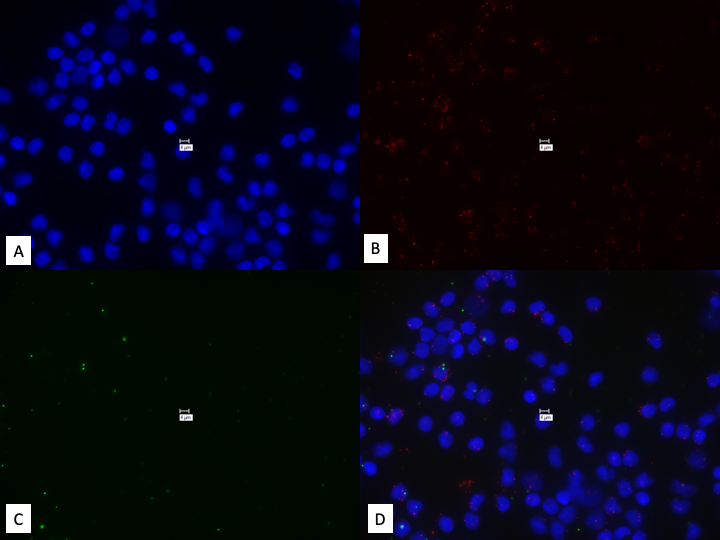

Supplement: Supplementary file 14 — Additional file 14. With probes: Human circulating buffy coat white blood cells. A = DAPI; B = Texas Red (Probe is I Human β-actin; Type 1 Red); C = Cy-5 Probe is IS 900 Type 6 Green) (D = composite of A, B and C. Note scattered apparent “positive “signal in panels “B”, “C” and “D”. Marker bars in µm indicates magnification of × 100. [file 13104_2020_4947_MOESM14_ESM.tiff]

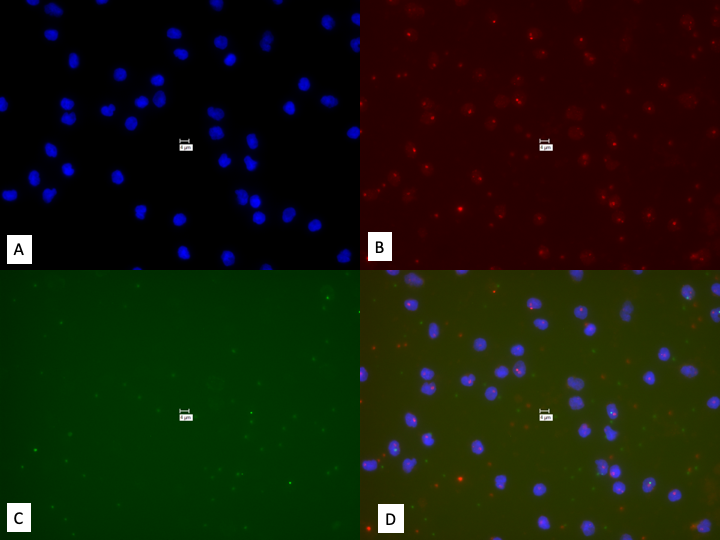

Supplement: Supplementary file 15 — Additional file 15. No-Probe control for Additional file 14. Human circulating buffy coat white blood cells. A = DAPI; B = Texas Red (No-Probe) C = Cy-5 No-Probe) (D = composite of A, B and C. Note scattered apparent “positive “signal in panels “B”, and “D”. Although less pronounced than in Additional file 14, false positive signal is detectable in this No-Probe control. Marker bars in µm indicates magnification of × 100. [file 13104_2020_4947_MOESM15_ESM.tiff]

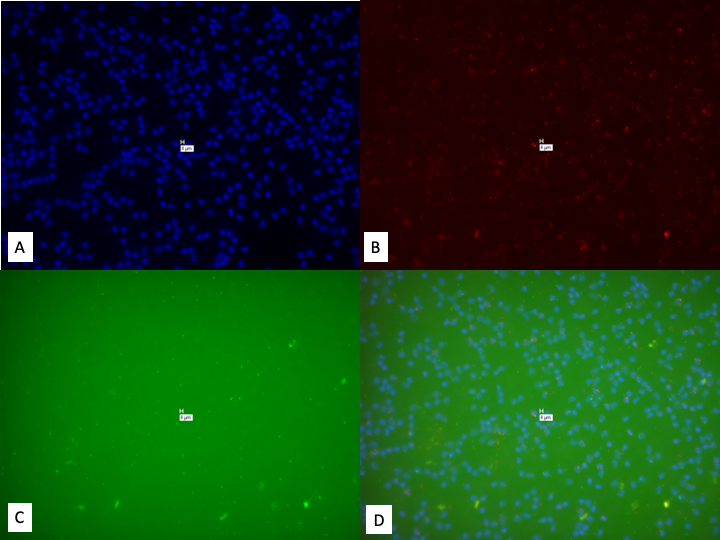

Supplement: Supplementary file 16 — Additional file 16. With probes: Drawn into DNA Paxgene® tubes. Human circulating buffy coat white blood cells. A = DAPI; B = Texas Red (Probe is I Human β-actin; Type 1 Red); C = Cy-5 Probe is IS 900 Type 6 Green) (D = composite of A, B and C. Note scattered apparent “positive “signal in panels “B”, “C” and “D”. Marker bars in µm indicates magnification of × 40. [file 13104_2020_4947_MOESM16_ESM.tiff]

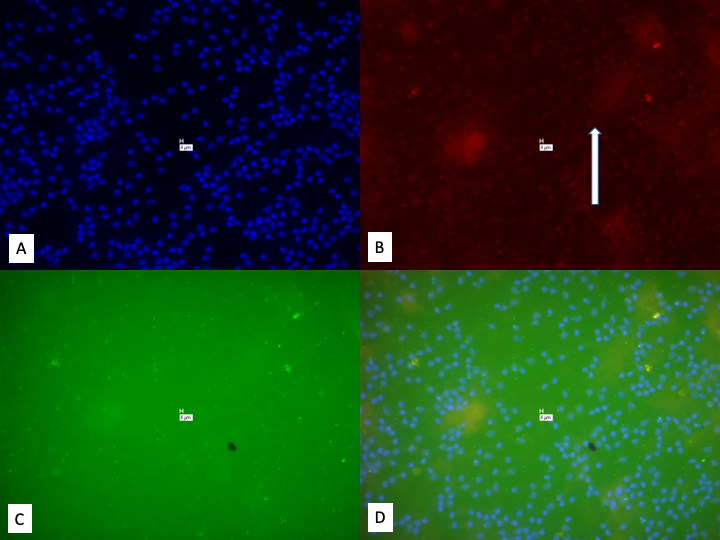

Supplement: Supplementary file 17 — Additional file 17. No-Probes control for Additional file 16: Drawn into DNA Paxgene® tubes. Human circulating buffy coat white blood cells. A = DAPI; B = Texas Red; C = Cy-5 (D = composite of A, B and C. Note scattered apparent “positive “signal in panels “B”, “C” and “D”. Arrow identifies false positive signal in Panel “B”. There is more abundant false positivity in “C”, Cy-5. Marker bars in µm indicates magnification of × 40. [file 13104_2020_4947_MOESM17_ESM.tiff]

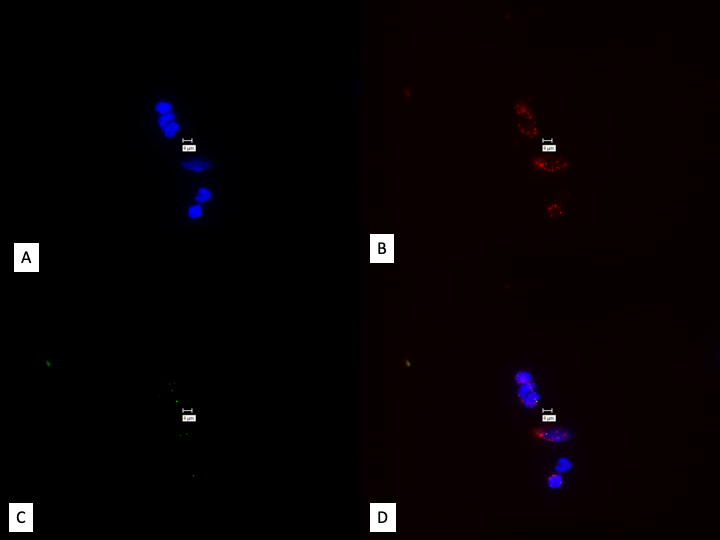

Supplement: Supplementary file 18 — Additional file 18. With probes: Human circulating buffy coat white blood cells. Specimens were processed immediately. A = DAPI; B = Texas Red (Probe is Human b-actin; Type 1 Red); C = Cy-5 Probe is Human GAPD (glycaraldehyde-3-phosphate dehydrogenase: Type 6 Green) (D = composite of A, B and C. Note scattered “positive “signal in panels “B”, “C” and “D”. These are always associated with DAPI positive regions, indicating that the signal is associated with white blood cells. They may be genuine and not be spurious background signal. Marker bars in µm indicates magnification of × 40. [file 13104_2020_4947_MOESM18_ESM.tiff]

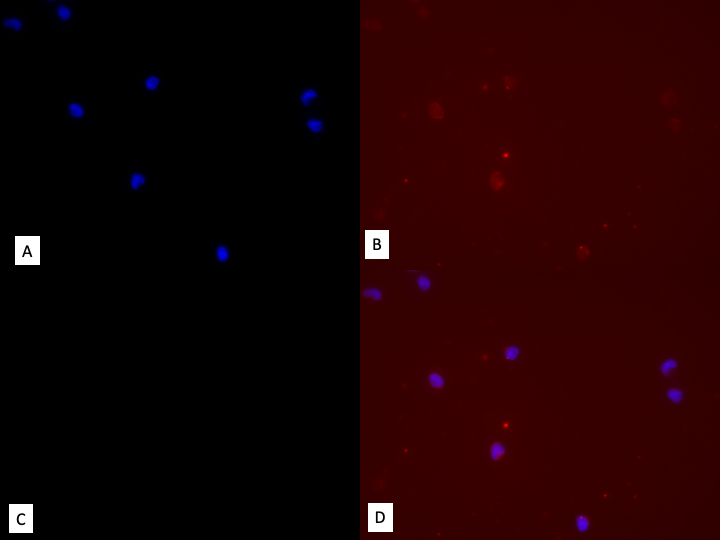

Supplement: Supplementary file 19 — Additional file 19. No-Probes control for Additional file 18: Specimens were processed immediately. Human circulating buffy coat white blood cells. A = DAPI; B = Texas Red; C = Cy-5 Probe (D = composite of A, B and C. Note scattered apparent “positive “signal in panels “B”, “C” and “D”. There is zero false positive signal with the Cy-5 filter (Panel “C”.) There is some signal associated with Texas Red (Panels B and D.) It is of significance that this, predominantly, is NOT associated with DAPI positive regions. This indicates that reliable negative control with circulating WBC’s may be achievable with the Affymetrix ViewRNA ISH Cell Assay Kit®; Invitrogen by Thermo Fisher Scientific, Catalog Number: QVC0001. Magnification is x 40. [file 13104_2020_4947_MOESM19_ESM.tiff]

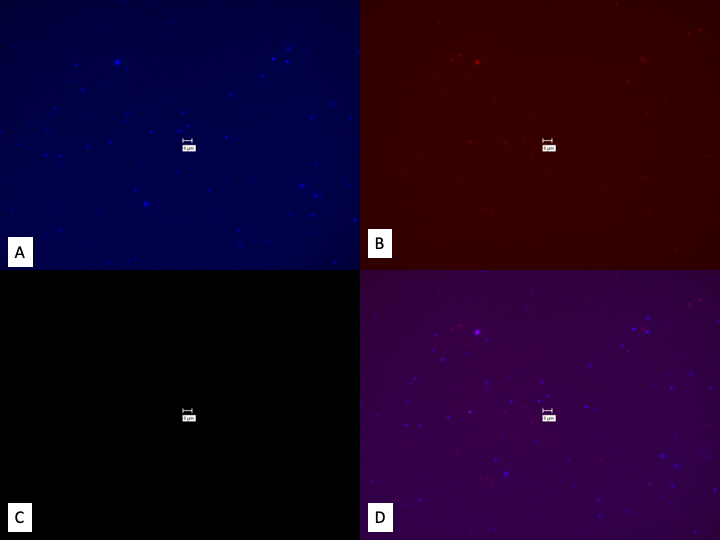

Supplement: Supplementary file 20 — Additional file 20. With probes: With RNA Later®. Specimens were stored at 4 °C for 24 h before being processed. Human circulating buffy coat white blood cells. A = DAPI; B = Texas Red (Probe is Human β-actin; Type 1 Red); C = Cy-5 Probe is Human GAPD (glycaraldehyde-3-phosphate dehydrogenase: Type 6 Green) (D = composite of A, B and C. Note scattered apparent “positive “signal in panels “B”, and “D”. Marker bars in µm indicates magnification of × 100. [file 13104_2020_4947_MOESM20_ESM.tiff]

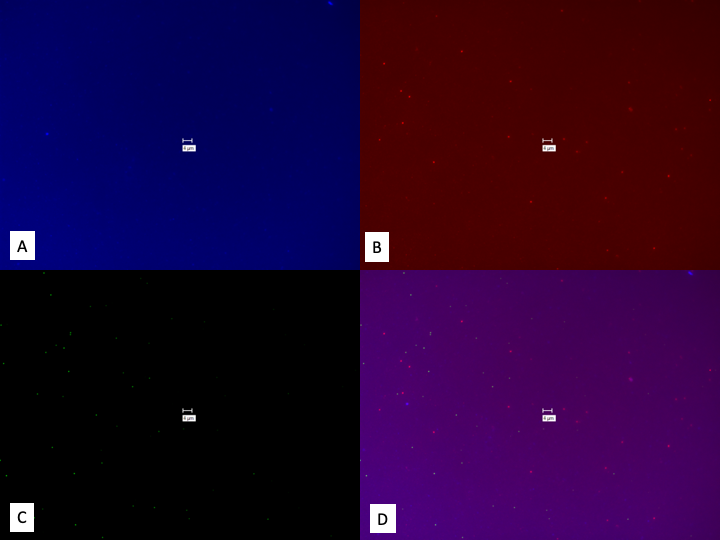

Supplement: Supplementary file 21 — Additional file 21. No-probe control for Additional file 20: With RNA Later®. Specimens were stored at 4 °C for 24 h before being processed. Human circulating buffy coat white blood cells. A = DAPI; B = Texas Red (Probe is Human β-actin; Type 1 Red); C = Cy-5 Probe is Human GAPD (glycaraldehyde-3-phosphate dehydrogenase: Type 6 Green) (D = composite of A, B & C. Note scattered apparent “positive “signal in panels “B”, “C” and “D”. Marker bars in µm indicates magnification of × 100. [file 13104_2020_4947_MOESM21_ESM.tiff]

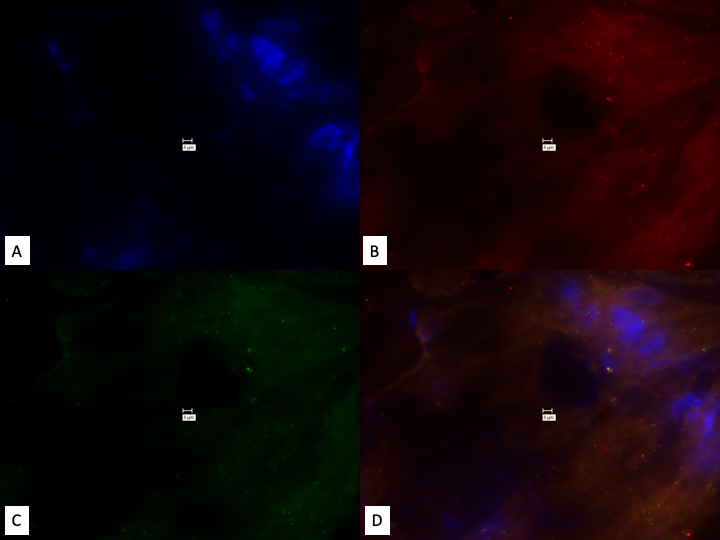

Supplement: Supplementary file 22 — Additional file 22. Affymetrix Single Cell Kit with Johne intestine. With Probes. A = DAPI; B = Texas Red (Probe is Bovine β-actin; Type 1 Red); C = Cy-5 (Probe is IS 900 Type 6 Green) (D = composite of A, B and C.) Purportedly positive signal is seen in panels “B”, “C” and “D”. Marker bars in µm indicates magnification of × 100. [file 13104_2020_4947_MOESM22_ESM.tiff]

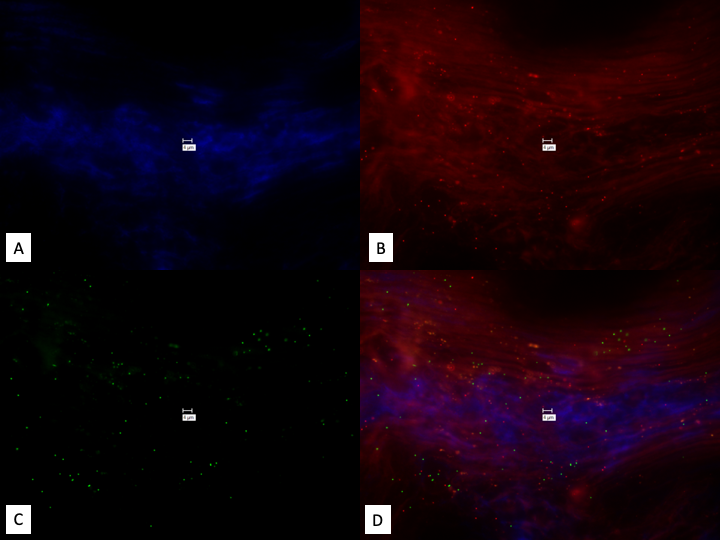

Supplement: Supplementary file 23 — Additional file 23. Affymetrix Single Cell Kit with Johne intestine. No-Probe control for Additional file 22. A = DAPI; B = Texas Red C = Cy-5 (D = composite of A, B and C.) Abundantly false positive signal is seen in panels “B”, “C” and “D”. This indicates that the Affymetrix Single Cell kit is of no utility studying archived frozen intestine. Marker bars in µm indicates magnification of × 100. [file 13104_2020_4947_MOESM23_ESM.tiff]

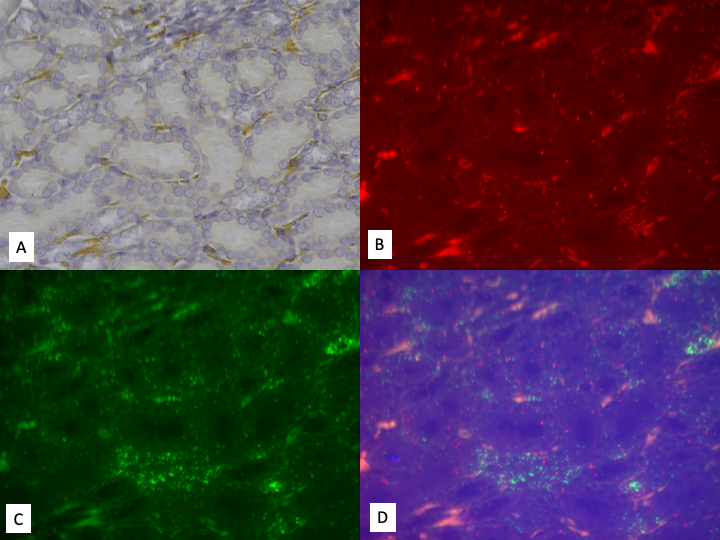

Supplement: Supplementary file 24 — Additional file 24. Affymetrix in house controls, provided to our laboratory. Slides were fully processed prior to arrival in our laboratory. The only action taken by us, was to read them. Panel A is Bright Field. Panels B = Texas Red C = Cy-5 (D = composite of B and C.) Tissues is rat fetal kidney. False positive signal is seen in panels “B” “C” & “D”. [file 13104_2020_4947_MOESM24_ESM.tiff]
